# Neuropathic Component Characteristics in Chronic Secondary Musculoskeletal Pain After Postmenopausal Osteoporotic Fractures: A Pilot Cross-Sectional Study

**DOI:** 10.1155/prm/9766698

**Published:** 2024-12-26

**Authors:** Marie-Eva Pickering, Serge Perrot, Dualé Christian, Véronique Morel, Nicolas Macian, Bruno Pereira

**Affiliations:** ^1^Rheumatology Department, CHU Gabriel Montpied, Clermont-Ferrand 63000, France; ^2^Pain Center, Cochin Hospital, INSERM U987, Paris Cité University, Paris, France; ^3^Clinical Investigation Center, PIC/CIC, University Hospital, CHU, Clermont-Ferrand, France; ^4^Biostatistics Unit DRCI, University Hospital, Clermont-Ferrand, France

**Keywords:** fracture, neuropathic pain, osteoporosis, treatment

## Abstract

**Background:** The neuropathic characteristics of pain occurring after an osteoporosis (OP)-related fracture are often under-recognized. The aim of this pilot study is to identify, in patients suffering from pain localized on the site of a previous osteoporotic fracture, the presence of neuropathic characteristics, their medical management, and their impact on quality of life.

**Methods:** This pilot cross-sectional study on consecutive patients in University Hospital, Rheumatology Department, Clermont-Ferrand, France, was approved by the Ethics Committee (IRB number 2023-CF34). Pain was evaluated with the Numeric Pain Rating Scale (NPRS), Neuropathic Component of Chronic pain (NCCP) was screened with the DN4 questionnaire, and sleep was assessed with the Pittsburg questionnaire. Depression, anxiety, quality of life, and concomitant treatment were also evaluated. Results were expressed using effect sizes (ESs) and 95% confidence intervals.

**Results:** Fifty new patients with a history of at least one fully documented fragility vertebral fracture (VF) or nonvertebral fracture (NVF) due to osteoporosis, in the last 2 years minus the previous 6 months, were included. Findings show that 21% patients with VF and 28% patients with NVF reported NCCP (DN ≥ 4). NCCP patients had more intense pain (NPRS = 5.1 ± 2.9 vs. 2.9 ± 2.7, ES = 0.82 [0.18; 1.44], *p*=0.019) and impaired sleep compared to patients without NCCP (ES = 0.71 [0.08; 1.33], *p*=0.043). A remarkable point was that patients had no specific oral or topical treatment for NCCP and were only taking on demand paracetamol and nonsteroidal anti-inflammatory drugs.

**Conclusions:** Future research should focus on the neuropathic characteristics of pain patients with OP, in order to better manage OP-related pain.

## 1. Introduction

Osteoporosis (OP) affects up to 30% of postmenopausal women and is characterized by loss of bone mineral density, deterioration in bone microarchitecture, increased risk of fragility fracture, and morbidity [1, 2] When a fracture damages the neat fiber meshing of bone architecture, it also disrupts the sensory nerve fibers at different levels of the bone structure, leading to pH changes and local acidosis, reinforced by the overactivity of osteoclasts and the release of algogenic products [3, 4]. A fracture usually heals over several weeks, but acute pain may become chronic with peripheral and central sensitization, involving an array of neurotransmitters, receptors (i.e., N-methyl-D-aspartate receptors (NMDARs)) [5], and cells (glial cells) and the modulation of descending pain pathways [6]. Chronic pain after osteoporotic vertebral fracture (OVF) is often reported by the patient as “back pain” or “low back pain” that may be invalidating, all the more since OP affects older persons who also suffer from other comorbidities [7]. Low back pain, with an estimated lifetime prevalence of 84% [8], is a major cause of disability [9]. An under-recognized neuropathic component in back pain in the general population has been underlined in the literature [10], and this also applies to vertebral OP since axial low back pain is traditionally presented as nociceptive [8, 11]. OP is often considered to be a silent pathology until a fracture occurs, but studies suggest that OP with and without fracture may be a painful pathology. Using the Pain-Detect Questionnaire (PD-Q) and looking for pain symptoms, a study revealed that without any vertebral fracture (VF), the percentage of osteoporotic patients with nociceptive back pain and neuropathic pain (NP) or mixed pain was 85% and 15%, respectively [12]. The presence of NP after an OVF [13] is not well defined, and data on NP management in osteoporotic fractures are still scarce. A recent Italian study [14] showed in patients with OVFs a prevalence of neuropathic components of chronic back pain ranging from 5.5% to 23.6%, according to the used tool, PD-Q and LANSS, respectively. According to the ICD 11 definition [15], the type of pain occurring with OP and osteoporotic fractures refers to chronic secondary musculoskeletal pain, and a neuropathic component may be present. The genesis of the neuropathic component of chronic secondary musculoskeletal pain (NCCP) is not clear yet. It may occur with nociceptive and nociplastic pain following damage to the bone, to nervous structures, and/or secondary to central or peripheral sensitization phenomena.

Characterization of the NP component in OP is important to consider, as it is associated with a deleterious impact on the quality of life and function. The aim of this pilot study is therefore to identify in patients suffering from pain localized on the site of osteoporotic fractures, vertebral but also nonvertebral, the occurrence of a neuropathic component, its medical management, and its impact on quality of life.

## 2. Methods

### 2.1. Participants

We conducted a pilot cross-sectional study, according to the STROBE checklist criteria [16]. The study was approved on March 6, 2023, by the Ethics Committee (IRB00013412, “CHU de Clermont-Ferrand IRB 1,” IRB number 2023-CF34) with compliance to the French policy of individual data protection. New patients referred to the Rheumatology Department by their General Practitioner were consecutively recruited for the management of their OP. They were informed about the study and provided informed consent to the processing of personal data. Inclusion criteria were a history of at least one vertebral or nonvertebral osteoporotic fracture confirmed by imaging over the last 2 years, but at least 6 months before inclusion. Exclusion criteria were acute pathology, acute back pain, and any other cause of back pain including chronic widespread pain or fibromyalgia.

### 2.2. Tests and Questionnaires

VFs were identified by conventional radiographs, morphometry analysis, and dual-energy X-ray absorptiometry (DXA). All patients had OP and attended the clinic for specific management of OP.

Pain was evaluated with the Numeric Pain Rating Scale (NPRS). This scale is an 11-point scale for patient self-reporting of pain. Patients verbally rated the intensity of their average and worst pain over the last week on a scale from “0” (no pain) to “10” (worst pain possible). Severe pain was > 6/10 on the NPRS. NP was screened with the “neuropathic pain in 4 questions questionnaire (DN4)” [17], a clinical tool for the diagnosis of NP. This questionnaire includes a series of four questions consisting of both sensory descriptors and signs related to bedside sensory examination. Two questions are based on the interview of the patient, and two are based on a standardized clinical examination. A score of 1 is given to each positive item and 0 to each negative item. The total score is the sum of the 10 items, and the cutoff value for the diagnosis of NP is a total score of 4/10.

Anxiety and depression were evaluated with the Hospital Anxiety and Depression Scale (HADS) [18]. This fourteen-item scale is used to identify possible and probable anxiety and depression disorders. Seven of the items relate to anxiety and seven relate to depression (high scores indicate more severe disease).

Sleep quality was evaluated with the Pittsburgh Sleep Quality Index (PSQI) [19]. This questionnaire with 19 items consists of 7 domains: subjective sleep quality, sleep latency, duration, disturbances, habitual sleep efficiency, use of sleep medication, and daytime dysfunction (high scores indicate poorer sleep quality).

Quality of life was evaluated with Short-form Health Survey SF-36. This questionnaire [20] is a measure of health status and quality of life of the patient. It includes eight health concepts divided into two categories, physical health and mental health. The physical health category includes general health perception, physical functioning, role limitations due to physical health problems, and bodily pain. The mental health category includes role limitations due to emotional problems, social functioning, mental health, and vitality. It also includes a single item that provides an indication of perceived change in health (higher scores indicate better health quality).

Body mass index (BMI) was calculated as the ratio of an individual's weight in kilograms divided by the height in meters squared (BMI = kg/m^2^).

### 2.3. Statistics

The sample size estimation was determined to guarantee a satisfactory statistical power for the analysis of neuropathic component effect on the quality of life of patients suffering from pain localized on the site of osteoporotic fractures. Sample size was calculated according to CONSORT Statement extension for randomized pilot and feasibility trials and Cohen's recommendations [21], defining effect size (ES) limits as small (ES: 0.2), medium (ES: 0.5), and large (ES: 0.8, “grossly perceptible and therefore large”). According to data reported in the literature and considering this study as a pilot, it seems suitable to include 50 patients in order to be able to highlight ES at least one standard deviation, with a type I error of 5% and statistical power greater than 80%.

Continuous data were expressed according to their statistical distribution with mean and standard deviation. The assumption of normality was analyzed using the Shapiro–Wilk test. The comparisons between groups (such as fracture with or without NP) were performed using Student's *t*-test or Mann–Whitney test if the assumptions of *t*-test were not met. The equality of variances was analyzed using the Fisher–Snedecor test. Concerning categorical data, the comparisons were conducted using chi-squared or Fisher's exact test. Statistical analyses were performed using Stata software (Version 15, StataCorp, College Station, USA). All statistical tests were carried out for a two-sided type I error at 5%. The results were expressed using ESs and 95% confidence intervals and were interpreted according to aforementioned Cohen's recommendations.

## 3. Results

**Figure 1:**
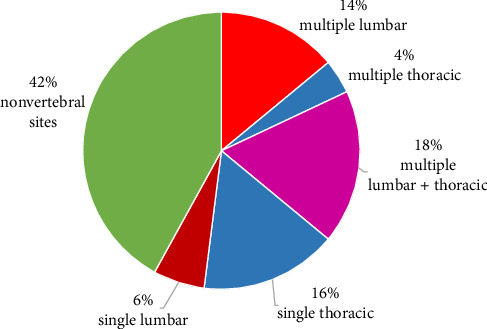
Sites of fragility osteoporotic fractures.

Fifty women with postmenopausal OP were included (Table 1). The mean age of the participants was 69.1 ± 7.7 years. Patients had OVFs (*n* = 29, 58%) or osteoporotic fractures on other locations (ONVF) (hip and wrist) (*n* = 21, 42%). OVFs were multiple (*n* = 18, 36%) or single (*n* = 11, 22%). The most common site of OVFs was lumbar spine, with single (*n* = 8, 16%) or multiple fractures (*n* = 7, 14%) (Figure 1).

Pain intensity in the total population was NPRS: 3.5 ± 2.7, and 14 patients had DN ≥ 4 (28%). Twelve patients (24%) displayed neuropathic characteristics with at least one point on the DN4 questionnaire. Concerning the 14 patients with DN ≥ 4, 6/50 patients had VFs and 8/50 had fractures in other locations: 12% had NP in OVF (6% lumbar and 6% thoracic). Out of 29 patients with an OVF, 6 patients had NP (21%). Patients with a neuropathic component had not received any of the recommended treatments for NP and had not been evaluated for suspicion or presence of NP previously.

Patients with NCCP had more pain (5.1 ± 2.9 vs. 2.9 ± 2.7, ES = 0.82 [0.18; 1.44], *p*=0.019), more severe pain (> 6) (64% vs. 33%, *p*=0.046), and impaired sleep (10.1 ± 4.7 vs. 7.2 ± 3.5, ES = 0.71 [0.08; 1.33], *p*=0.043). However, SF-36 physical (ES = −0.11 [−0.72; 0.50]) and mental dimensions (ES = −0.29 [−0.90; 0.32]), anxiety (ES = 0.30 [−0.31; 0.91]), and depression (ES = 0.26 [−0.36, 0.86]) were not significantly different according to fracture with NP component/characteristics.

There were no significant differences between OVF and ONVF groups for age, BMI, pain, quality of life scores, anxiety, depression, and sleep and no significant differences between patients with or without NP for BMI, quality of life, and depression.

Patients were taking over-the-counter analgesics as needed: paracetamol (30%) and nonsteroidal anti-inflammatory drugs (25%). Only one patient was on antidepressant (venlafaxine) for anxiety symptoms. None was on anticonvulsants, antidepressants, or topical patches (lidocaine 5% and capsaicin 8%) for NP, as recommended in guidelines.

## 4. Discussion

This pilot study in postmenopausal women with fractures shows that 28% patients exhibit NCCP at nonvertebral site and 21% patients at vertebral site. These findings confirm results from a recent study [14] that showed a prevalence of up to 23.6% NP using LANSS questionnaire, one of the three commonly used tools with PD-Q and DN4 to detect the presence of NP components [22]. In our study, we used the DN4 questionnaire in French, the language in which it was first validated, and its psychometric properties to assess NP were confirmed in patients with low back pain [23]. This is important as many tools have been translated and not revalidated in another language or clinical setting and might bias NP assessment [8]. However, the DN4 is not specific to NP of bone fracture origin and the use of this metric may be confounded by NP that is present from other origins, although we were careful to check this was not the case. DN4 only defines the presence or absence of neuropathic characteristics, not evaluating mixed pain unlike other tools such as the PD-Q. In the future, it could be interesting to explore the relationship between these tools further. The study also shows that patients with the NP component had more pain, more severe pain, and impaired sleep compared to patients with no NP. Concerning the localization of the VF, the sample of our pilot study was not large enough to identify differences between dorsal and lumbar locations as regards the severity and quality of pain and other measured parameters. This aspect should be considered in a future study in order to identify the impact of the localization of the fracture on a patient's clinical condition, mobility, and quality of life.

Fragility fractures in OP, often revealed by acute pain, have a large impact on the quality of life of patients as they may lead to chronic pain of variable intensity, duration, and characteristics, including NP. Complaints on back pain are however rarely attributed to OP itself [24], and OP fracture as an etiology of back pain is often overlooked. Interestingly, the presence of a NP component in our patients was new to them, as they had never been assessed for NCCP previously nor treated with recommended NP treatments before their outpatient specialist visit. This observation calls for additional recommendations to present guidelines especially on back and low back pain, as these often do not specify OP consequences as a possible cause of NP development that is still under-recognized in OP patients.

This pilot study was well powered in order to highlight large ESs that have been verified for the relationships between pain, impaired sleep, and NP. Parameters of quality of life including depression and anxiety, although often described in NP patients, do not show, in this work, significant magnitude of difference with small to mild ESs less than 0.3. Parameters of quality of life including depression and anxiety, although often described in NP patients, do not reach significance probably because of the small sample size of the study, and larger samples should be included in future trials in order to obtain larger subgroups. The study however shows that sleep is significantly affected by the presence of NP. Pain is known to disrupt sleep, lack of sleep increases pain perception [25], and sleep disturbances are particularly troublesome in patients with painful rheumatic disease [26]. Sleep efficiency (NREM sleep) [27, 28] and quality of life are reduced in NP [29]. Although the mechanism of this bidirectional association between sleep disturbances and NP remains unclear, the opioidergic, dopaminergic, immune systems as well as orexins, adenosine, and the hypothalamic–pituitary–adrenal axis are known to be involved.

In conclusion, this pilot cross-sectional study showed that NCCP is present in some patients after OP fragility fracture and impairs their sleep. Diagnosis and treatment of NCCP occurring in the context of OP need to be implemented earlier in the care journey. Future research on studies with larger samples also needs to focus on the characteristics and prognosis of NCCP in patients with OP in order to better understand and manage it and assist patients for pain alleviation and better sleep and everyday life quality.

## Figures and Tables

**Table 1:** Characteristics of patients and the presence of neuropathic pain (NP).

	Fracture with no NP	Fracture with NP	*p* value
*n*	21	29	
Age	70.6 (7.4)	65.2 (6.9)	0.04
BMI	25.1 (9.4)	22.5 (5.0)	0.09
PSQI	7.2 (3.5)	10.1 (4.7)	0.04
SF-36 physical	42.9 (11.8)	41.6 (9.2)	0.70
SF-36 mental	46.9 (10.1)	43.7 (12.1)	0.50
HADS depression	5.0 (3.5)	5.9 (3.0)	0.48
HADS anxiety	7.5 (2.8)	10.3 (6.6)	0.29

*Note:* Values are expressed as mean (standard deviation).

## Data Availability

The data used to support the findings of this study are available from the corresponding author upon reasonable request.
